# Supplementary material for: Clinicopathological Significances of Tumor–Stroma Ratio (TSR) in Colorectal Cancers: Prognostic Implication of TSR Compared to Hypoxia-Inducible Factor-1α Expression and Microvessel Density
Source: Curr Oncol. 2021 Mar 22;28(2):1314–24. doi: 10.3390/curroncol28020125 (PMC8025820; doi:10.3390/curroncol28020125)
Supplement: Supplementary file 1 [file curroncol-28-00125-s001.pdf]

Supplementary Materials

Clinicopathological Significances of Tumor–Stroma Ratio (TSR) in Colorectal Cancers: Prognostic Implication of TSR Compared to Hypoxia-Inducible Factor-1α Expression and Microvessel Density

Guhyun Kang, Jung-Soo Pyo, Nae-Yu Kim and Dong-Wook Kang

Table S1. The correlation between high microvessel density and overall and recurrence-free survivals in various subgroups according to the tumor stroma ratio.

Subgroups	Overall Survival (*p*-Value)	Recurrence-Free Survival (*p*-Value)
≥stroma 30%	0.132	0.297
<stroma 30%	0.458	0.458
≥stroma 50%	0.210	0.339
<stroma 50%	0.866	0.820
≥stroma 70%	**0.004**	**0.005**
<stroma 70%	0.053	**0.042**

$p < 0.05$ are highlighted in bold.

© 2021 by the authors. Licensee MDPI, Basel, Switzerland. This article is an open access article distributed under the terms and conditions of the Creative Commons Attribution (CC BY) license (http://creativecommons.org/licenses/by/4.0/).
